# A Role for the Cytoskeleton in Heart Looping

**DOI:** 10.1100/tsw.2007.87

**Published:** 2007-02-19

**Authors:** Kersti K. Linask, Michael VanAuker

**Affiliations:** ^1^ University of South Florida (USF) and All Children's Hospital (ACH), Department of Pediatrics, The Children's Research Institute, St. Petersburg, FL 33701, USA; ^2^ University of South Florida (USF) and All Children's Hospital (ACH), Department of Chemical Engineering, Tampa, FL 33620, USA

**Keywords:** heart looping, cardiac, nonmuscle myosin II, flectin, cytoskeleton, actin, biomechanics, blood flow, shear stress, integrin, microcilia, extracellular matrix

## Abstract

Over the past 10 years, key genes involved in specification of left-right laterality pathways in the embryo have been defined. The read-out for misexpression of laterality genes is usually the direction of heart looping. The question of how dextral looping direction occurred mechanistically and how the heart tube bends remains unknown. It is becoming clear from our experiments and those of others that left-right differences in cell proliferation in the second heart field (anterior heart field) drives the dextral direction. Evidence is accumulating that the cytoskeleton is at the center of laterality, and the bending and rotational forces associated with heart looping. If laterality pathways are modulated upstream, the cytoskeleton, including nonmuscle myosin II (NMHC-II), is altered downstream within the cardiomyocytes, leading to looping abnormalities. The cytoskeleton is associated with important mechanosensing and signaling pathways in cell biology and development. The initiation of blood flow during the looping period and the inherent stresses associated with increasing volumes of blood flowing into the heart may help to potentiate the process. In recent years, the steps involved in this central and complex process of heart development that is the basis of numerous congenital heart defects are being unraveled.

## INTRODUCTION

Heart looping is a central process in cardiac development. Looping in all vertebrates that have been analyzed begins with a rightward movement of the anterior part of the developing straight heart tube. At this period, the ventricular area of the heart is cephalad to the atrial region. Blood flow into the straight heart tube is initiated concomitant with initiation of myocardial contractions and of looping. Looping is completed with the positioning of the atria anterior to the ventricles and dorsal to the outflow tract. Only after completion of looping does the four-chambered heart form. Subsequent heart development involves formation of the septa and valves, thus leading to parallel blood flow through the heart. Changes in gene expression or protein interactions that are involved in this complex process have serious consequences for subsequent heart development. It is thought that many cardiac anomalies associated with congenital heart disease arise with this process.

## LATERALITY AND DIRECTIONALITY OF CARDIAC LOOPING

The heart is the first organ to break left-right symmetry in the embryo by looping in a rightward, or dextral, direction. Therefore, cardiac looping direction has been widely used as an experimental read-out for defining genes that specify laterality in the embryo[1,2,3,4,5,6]. Many complex human congenital heart defects occur when genes associated with laterality specification in the embryo have mutated or been deleted[7]. Although the heart may be the first organ to break left-right axis symmetry, an earlier process associated with establishment of laterality was shown to involve nodal flow that is autonomously generated by the leftward movement of cilia in a 9+0 configuration that are tilted caudad on cells of the ventral node[8,9,10,11]. Recent evidence suggests that the leftward movement of membrane-sheathed particles is associated with the activation of the noncanonical Hedgehog signaling pathway and results in an asymmetric elevation of intracellular $Ca^{++}$, together with changes in gene expression[8,12,13,14]. Nodal flow, however, is not conserved in vertebrates and it may not be the only mechanism that breaks asymmetry in the mouse. For example, we observed an asymmetric expression of the sodium-calcium exchanger NCX-1 protein with greater expression apparent in the right side of Hensen's Node than on the left[15]. It was suggested that calcium signaling, as based on the asymmetric NCX localization in the Hensen's Node, similar to that of connexin 43[16], may affect laterality[15]. In the frog and avian embryo, ion channels and gap junction communication have been shown to have roles in establishment of asymmetry[17]. Downstream from the nodal flow/gap junction/NCX-1 asymmetry is the activation of *Nodal* gene expression. Nodal expression in vertebrates was usually reported as localizing only in the left lateral plate mesoderm (LPM) and not in the right. Recent experimental data using RT-PCR and mathematical modeling indicate, however, that Nodal expression is seemingly initiated in the right LPM by an activating signal. Right LPM *Nodal* expression is then rapidly down-regulated by inhibition from the midline, and remains expressed in the left LPM by what the authors term a "self-enhancement and lateral-inhibition" system (SELI)[18]. The resulting left LPM *Nodal* expression precedes visible heart looping or axial rotation of the embryo. It remains expressed until approximately the 12–14 somite stage[19,20]. Downstream of *Nodal* is *Pitx2,* a homeobox gene that is the only laterality gene shown so far to be expressed within the left wall of the straight heart tube and not in the right. Deletion or inactivation of *Pitx2* has been correlated with defects in heart morphogenesis: The observed developmental anomalies include valve defects, sinoatrial anomalies, and alignment problems, as characterized by double outlet right ventricle and transposition of the great arteries, as well as right ventricle hypoplasia[21,22,23]. It should be noted that Pitx2 *null* mice had correct, dextral looping of the heart. Our misexpression studies of *Pitx2c* and *CFC* in the chick heart fields provided evidence that expression of a specific threshold concentration of flectin, a novel myocardial-associated protein in the left or right chick embryonic heart, was the best indicator of the direction and rotation of heart looping[24,25,26,27]. Other reports on the importance of threshold levels of Pitx2 activity in the mouse[22] supported this conclusion.

## THE SECOND HEART FIELD AND A ROLE FOR THE DORSAL MESOCARDIUM IN HEART LOOPING

The heart tube during looping includes the following segments in anterior to posterior order: the developing outflow tract and part of the right ventricle that are emerging from the anterior heart field, the left ventricle that has formed from the first cardiac lineage and displays a C-loop at this time, and the right and left atria forming at the same posterior level from the posterior heart field[28]. Thus, looping occurs concomitantly with an increase in length of the primary or primitive heart tube with new segments being added from the second, anterior and posterior, heart fields. Anteriorly, the formation of the outflow tract occurs by asymmetric cell proliferation. There are more mitotic cells in the left dorsal mesocardial fold, and in the ventral floor of the foregut than in the right fold[29] (Fig. 1). We suggested that it is this asymmetric, left-dominant, cell proliferation pattern that drives the dextral directionality of looping. Experimental evidence

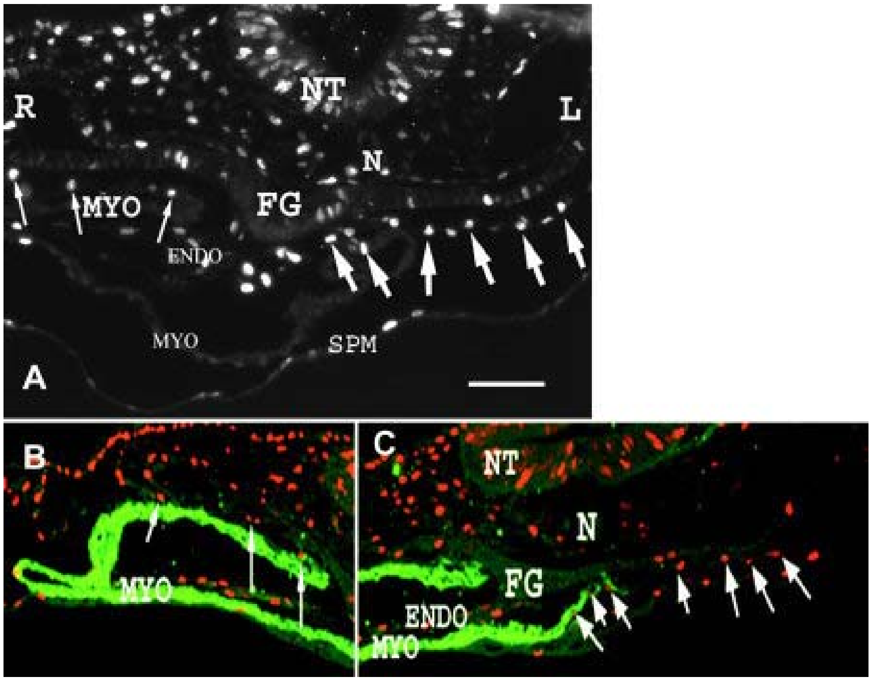

**FIGURE 1.** Asymmetric cell proliferation is evident in the dorsal mesocardial (DM) folds that are emerging from the anterior heart field. BrdU-labeling (arrows) shows that more cells are undergoing mitosis in the left DM fold (larger arrows) than in the right (smaller arrows) to initiate rightward directionality of looping between 7–9 somite stages. Reproduced with permission from Linask et al.[29].

indicates that the dextral direction, as based on signaling associated with asymmetric cell proliferation patterning in the chick, occurs between the 7–9 somite stage, i.e., early HH stage 9[29]. Posteriorly, the right and left atrial segments are forming by addition of cells to the primary heart tube in a symmetric manner. As cells arise from the second lineage contributing to the length of the heart tube[28], the dorsal mesocardial (DM) folds also form from the anterior field. The DM folds continue to display asymmetric cell proliferation[29]. The left and right DM folds approach each other from the lateral regions, merge, and then constrict at the embryonic midline. The dorsal mesocardium maintains the position of the heart relative to the midline of the ventral floor of the foregut. As looping proceeds, the elongating heart tube deepens its rightward bend.

In order for the heart tube to continue bending or looping, it must be released from its DM attachment along most of the heart tube. The DM attachment undergoes relatively rapid degradation by matrix metalloproteinase (MMP) activity, first apparent at the mid-level of the heart tube. The breakdown by MMP-2 activity continues anteriorly and posteriorly until the heart remains attached posteriorly only at the anterior intestinal portal. This posterior attachment site becomes the permanent DM (PDM) attachment area at the midline of the embryo[29]. The PDM is characterized by being rich in several extracellular matrix (ECM) proteins and is seemingly protected from proteolytic breakdown, as no MMP-2 mRNA is detected at this site. Apoptosis is not involved in the dissolution of the dorsal mesocardium and the breakdown occurs by degradation of the ECM[29]. It is only after the breakdown of the rest of the dorsal mesocardium that heart tube looping can be completed. It is noteworthy that MMP-2 activity is involved in coordinating several aspects of heart morphogenesis: first, the fusion of the right and left cardiac epithelial compartments to form a tube; subsequently, in directionality of looping through effects on asymmetric cell proliferation; and finally, enabling the completion of looping with the breakdown of the dorsal mesocardium. Mechanisms of MMP-2's role may relate to activation of factors at the membrane or the release of growth factors or growth factor-like domains from the ECM that can then bind to cell surface receptors. The precise definition of the MMP mechanism remains among areas for future investigation.

## THE UNDERLYING CELLULAR MECHANISMS OF HEART LOOPING

The shaping of tissues during organ development requires cell migrations, cell shape changes, and bending of epithelial sheets to form tubular structures that, in turn, undergo subsequent morphogenetic movements until the final shape of the organ is achieved[30,31]. These described morphogenetic changes all occur within the context of early heart development. Mesenchymal precardiac cells migrate from the anterior regions of the primitive streak into the bilateral heart fields[32,33]. The precardiac mesenchymal cells migrate anteriorly and toward the midline over a fibronectin substratum[34,35,36,37,38] where they sort out via N-cadherin/β-catenin–mediated adhesion to form two ventral, epithelial compartments[30,39,40]. The epithelial compartments bend ventrally and undergo ventral closure to form a straight heart tube[29,41]. The anterior-posterior and dorsal-ventral patterning of cell-cell and cell-matrix adhesion molecules and their activity in signal transduction pathways are keys to driving the three-dimensional form of the organ, as well as cell differentiation. It is now becoming generally appreciated that the cross-talk between these adhesion signaling pathways associated with cell differentiation and heart morphogenesis involves outside-in and inside-out signaling via the cytoskeleton (Fig. 2).

The information on the underlying molecular mechanisms of the bending and rotational component of looping remains very limited. From analyses of invertebrate models, it appears that the unconventional myosins in association with the actin cytoskeleton has a central role in left-right determination[42], and we suggest that the actomyosin cytoskeleton also have a central role in vertebrate heart looping. Cytoskeletal restructuring is required for cell shape changes, for the movement of cells, and for the shaping of tissues throughout morphogenesis. Cells depend on cytoskeletal molecules and molecular motors to establish their asymmetrical shapes, to transport intracellular constituents, and to facilitate cell motility. The assembly of the cytoskeletal components and action of the associated motors is largely responsible for establishing cellular architecture and tissue structure[43].

The bending forces within the heart tube appear to have a common underpinning related to tension and contractility of the cytoskeleton. Prior and recent studies have indicated actin to be a major component of looping[44,45,46,47]. Confocal laser scanning microscopy reveals that development and organization of actin filaments considerably differ in inner and outer myocardial cell layers[48]. Myocytes in the outer layer facing the pericardial cavity are round. In the inner layer, myofibrils localize at the bottom, facing the cardiac jelly. At the tubular heart stage when the tube begins to bend, F-actin is aligned with its long axis coinciding circumferentially in relation to the tubular structure (diagrammatically shown in Fig. 2), as also can be seen in this embryonic chick heart immunostained for actin localization and prepared for three-dimensional analyses (Fig. 3). It was suggested that these circumferentially arranged actin filaments at the base of the inner layer of the myocardium are important for promoting cardiac looping[48,49]. Fibronectin (FN) is aligned parallel with the actin bundles early in looping, but during looping, FN becomes more fragmented and appears in a speckled pattern[50]. We observed the speckled FN localization during looping as well[27]. This may relate to MMP-2 activity that we observed to be an important component of looping[29]. A member of a the Ca++-dependent family of adhesion molecules, N-cadherin, is responsible for the connection of actin and myofibrils between neighboring myoctes at cell-cell junctions[39,51,52], and for the cell alignment and arrangement of the two layers of the developing heart tube[53]. Loss of N-cadherin leads to alterations in connexins, possibly via changes in cytoskeletal signaling[54]. Additionally, it has been shown that N-cadherin is involved in the oriented responses of cardiomyocytes induced by mechanical stretch *in vitro*[55]. Thus, during looping, as in the earlier period of cardiac compartment formation, cross-talk between two mechanosensing and adhesion mediated-signaling pathways, integrin and N-cadherin, is evident[56]. The cross-talk appears to be facilitated by the cytoskeletal molecules as NMHC-II proteins and actin (see below), among others.

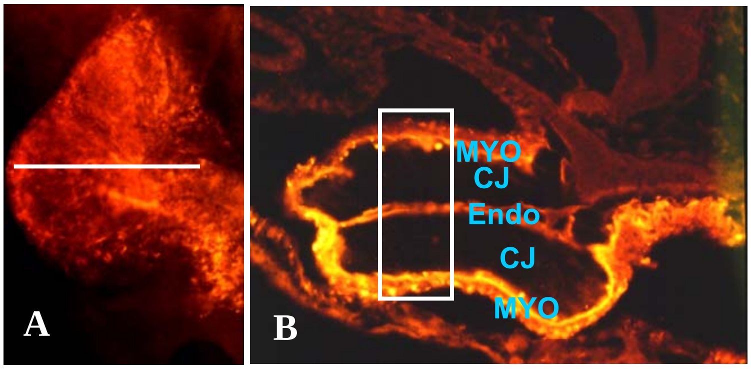

**A and B**

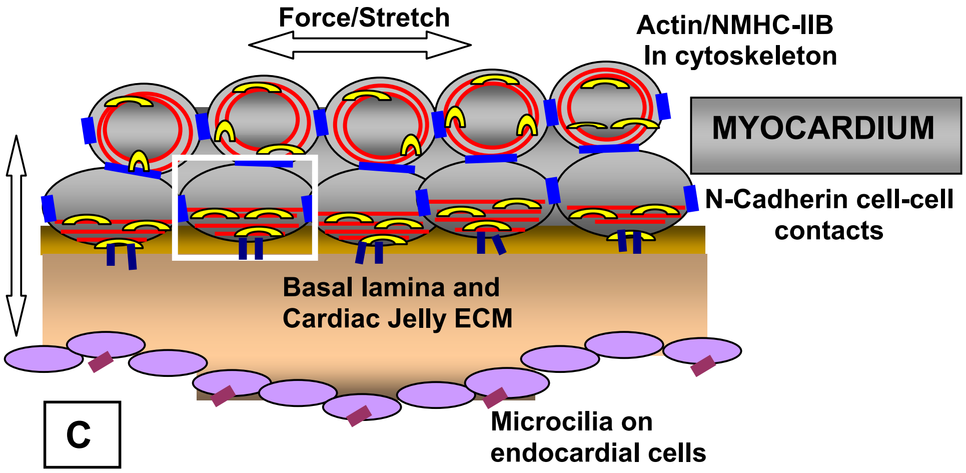

**C**

**FIGURE 2.** A hypothetical model based on current literature shows the cytoskeleton at the "hub" of cross-talk between cell-to-cell and cell-to-matrix interactions that are involved via signaling pathways in coordinating development of cardiac form and function. Accumulating evidence suggests that the cytoskeletal signaling involves interactions between the nonmuscle myosin II (NMHC-II) class of motor proteins and actin. Fig. 2A depicts a looping stage 11 chick heart immunostained for flectin. Section (plane of section depicted by white line) through the heart is shown in panel 2B showing the relationship (see boxed in region) of the myocardial (Myo) wall, basal lamina/cardiac jelly (CJ), and endocardium (Endo). Fig. 2C shows a diagram of a general model depicting the organization of the actin[48] (red fibers) and nonmuscle myosin (yellow arcs) in the inner and outer layer of the two-layered myocardium during the looping stages 11/12 in the chick heart. Basal lamina and cardiac jelly are shown, as well as the monociliated endocardial cells that are able to detect shear stresses of blood flow. Block arrows depict stretch and force on the cardiac cells.

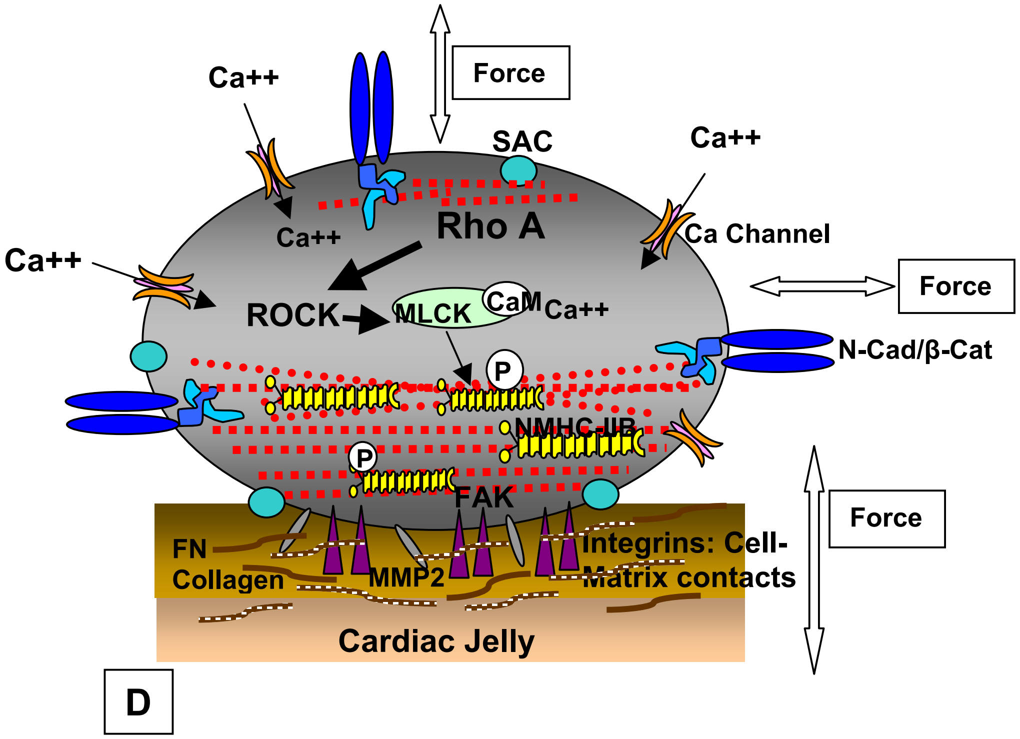

**FIGURE 2D**

**FIGURE 2 cont.** Panel 2D, depicts the subcellular cytoskeletal-mediated mechanisms in the inner myocardial wall by which focal adhesion kinase (FAK) at the basal side mediates integrin-, and its role in cyclic stretch, signaling in association with the cytoskeleton. Small cardiomyocyte cell processes are shown extending into the basal lamina (see also Fig. 6). MMP-2 is thought to interact within the ECM in the microenvironment to release activating factors that can bind to cell surface receptors, as well as lead to ECM degradation. N-cadherin/β-catenin complex functions in association with connexins and regulates cyclic stretch by its interactions with the cytoskeleton, as well as neighboring cells through mediation of cell-cell adhesion. Rho proteins, ROCK, Ca++/calmodulin (CaM), regulate force through phosphorylation of nonmuscle myosin light chain via myosin light chain kinase (MLCK), as well as phophorylation of other cytoskeletal proteins. In addition to the depicted calcium channels shown are stretch activated channels (SAC) that are known to be expressed in the embryonic chick heart[119]. Cation selective (Ca++ and Ba++) SACs can provide mechanosensitivity when a cell is mechanically stimulated and are proposed to be anchored to the cortical cytoskeleton and possibly to the ECM[120].

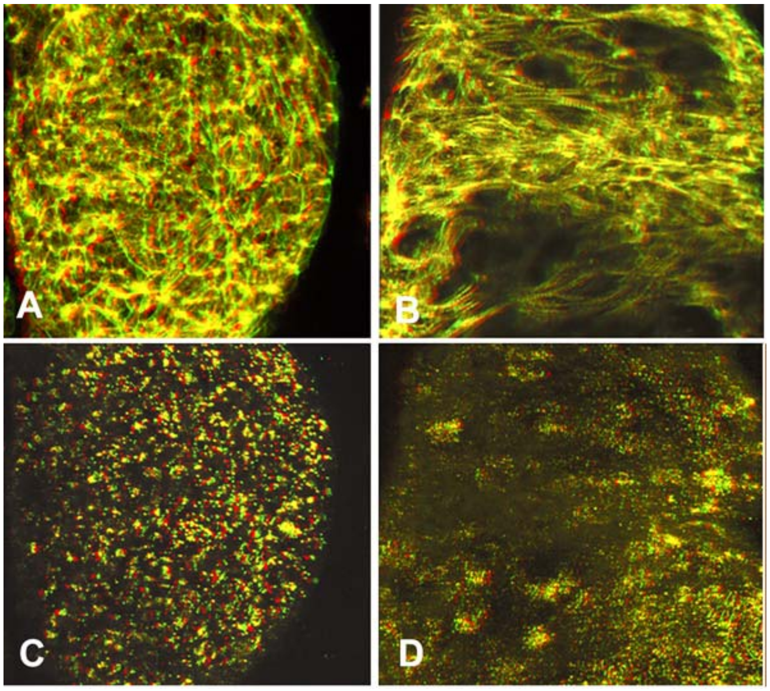

**FIGURE 3**. Three-dimensional confocal rendering of organization of F-actin (red, panels 3A, 3B) and flectin (green, panels 3C,3D) at HH stage 9 (Figs. 3A and C) and stage 11 chick heart (Figs. 3B and 3D). Readers will need red and green color glasses to visualize the 3D information. Between stages 9 and 11, F-actin in the myocardial layer near the cardiac jelly becomes circumferentially arranged around the heart tube during looping. More flectin is present in the left side of the avian heart tube in a punctate manner in relation to the myocardial layer and within the adjacent cardiac jelly.

## FLECTIN, NONMUSCLE MYOSIN II, AND LOOPING

Flectin was the first protein shown to be expressed in a left-right manner in the myocardium of the heart[27]. Flectin was identified as F22, a 240 kD protein [57,58]and was first described in relation to retinal pigment epithelial cells and the interphotoreceptor matrix (IPM) of the embryonic day 14 chick eye. It is an evolutionarily well-conserved protein[59]. The IPM is situated between the photoreceptors and the retinal pigment epithelium. It is involved in the development and maintenance of photoreceptors, and is a major factor in retinal adhesion[60]. Flectin is expressed asymmetrically in the myocardial wall of the mouse, as well as chick, embryonic tubular heart. There is dominant expression in the left heart wall and a lower level in the right. The dominant sidedness is modulated by laterality specifying genes. Regardless of the sidedness of Pitx2 expression, it is the dominant sidedness of flectin expression that most closely correlates with the direction of looping: If there is more flectin expressed in the left dorsal mesocardium and myocardium, hearts loop normally to the right; if there is more in the right side, hearts loop abnormally to the left. If there is no flectin or equal amounts of flectin in the two sides, the straight tubular heart does not loop[24,25].

In a recent collaborative study using immunoprecipitation with flectin antibody of looping heart extracts, flectin was identified by MALDI-Mass Spectroscopy and proteomics as closely related to, associated with, or being a variant of the nonmuscle myosin II (NMHC-II) family of proteins[61]. The precise separation and definition of what appears to relate to the nonmuscle myosin II isoforms are currently being analyzed. As based on available published data, in itself the nonmuscle myosin II protein family appears a good candidate to be involved in looping. The expression pattern of NMHC-IIB in the mouse heart appears similar to flectin in that a left-dominant asymmetric expression occurs in the dorsal mesocardial region and myocardium (unpublished observation). In the chick NMHC-IIB was shown immunohistochemically to be associated with actin during myofibrillogenesis in the differentiating cardiomyocyte[62]. A unique NMHC-IIA-like isoform has been described for retinal pigment bovine epithelial cells that exists in association with stress fibers and also in membrane vesicles[63]. NMHC-II proteins are well situated to act in mechanotransduction of biomechanical forces that are associated with initiation and increase of blood flow and the resulting compressive forces exerted on the cardiac jelly during the initiation of heartbeats, as well as of tube bending and rotational forces. Notably nonmuscle myosin IIs are found in many systems associated with mechanotransduction, for example in microcilia of the inner ear and in cell processes associated with afferent and efferent kidney arterioles[64,65,66,67]. When mutated, hearing loss and hypertension can result.

In early reports[26,27] we reported flectin as being only an asymmetrically expressed extracellular matrix (ECM) molecule due to its localization in the cardiac jelly, an extensive acellular region prevalent during the heart looping stages that is situated between the myocardium and endocardium. However, after degradation of the cardiac jelly using hyaluronidase, it was clear that flectin was present also intracellularly within the myocardium, and that it was modulated in the myocardial wall by upstream laterality genes[25,68]. It was, however, the dominant left-sidedness of flectin within the myocardial wall that most closely related to right heart looping. A role for the cardiac jelly ECM in looping can be questioned in general, because when the cardiac jelly is removed by hyaluronidase degradation of proteoglycans within this extracellular matrix compartment, both in the rat[69] or chick[68] embryos, the heart tube flattens, but continues to bend. It should be noted, however, that the glycoproteins within the myocardial basal lamina, as FN and collagen, most likely would remain localized, but was not analyzed in these experiments. Whether looping can be completed, remains to be established because the experiments were terminated too early. It has been claimed that the chick heart <u>isolated</u> in culture can still bend[70], also when blebbistatin that affects nonmuscle myosins is used to perturb cytoskeletal interactions[71]. However, the latter observation did not take into account that isolated tissues placed into culture tend to bend normally. It was not ruled out that the observed tissue "bending" may be due to a release of normal tensions on the myocardial tissue upon isolation. Importantly, in isolation the heart tube does not <u>complete</u> looping to form a four-chambered heart. We suggest that both normal <u>fluid stresses</u> and <u>asymmetric forces</u> within the myocardium are necessary to drive looping to completion.

Knockout mouse models for both NMHC-IIA and NMHC–IIB have been generated. The NMHC-IIA knockout embryo is an embryonic lethal already at ED 7.5, before a tubular heart forms[72]. In NMHC II-B knockout mice in which heart do develop, heart defects apparently associated with looping abnormalities are reported[73]. Approximately 65% of the NMHC-B-/- embryos died before birth, and those that were born had congestive heart failure and died during the first day after birth. Six of seven NMHC-IIB -/- newborn mice showed structural cardiac defects. These studies indicate that nonmuscle myosin II-B is required for normal cardiac development and that its absence results in structural defects associated with misalignment and in that in many respects, resemble two common human congenital heart anomalies, tetralogy of Fallot and double outlet right ventricle[73]. The absence of NMHC-IIB resulted in a significant increase in the transverse diameters of the cardiac myocytes. This may result in abnormal feedback of the developing myocardium, which in turn is unable to complete the cardiac looping process normally. There is a need to study the role(s) of NMHC-IIB, and its relationship with NMHC-IIA during heart tube formation and looping in still greater detail. In most systems where both nonmuscle myosins are expressed, the two NMHC-IIs have different roles within the same cell. Interestingly, nonmuscle myosin has been demonstrated as involved in promoting the retention of homeodomain transcription factors to the cytoplasm[74]. It would be of interest to determine whether a similar interaction occurs between Pitx2c and dominant- left sided NMHC-IIB expression in the heart.

## RHOA AND INTEGRIN SIGNALING PATHWAYS INVOLVEMENT IN CARDIAC FORM AND FUNCTION

In mammalian cells, Rho proteins regulate the formation of the actin cytoskeleton in stress fibers, lamellipodia, and filopodia and are downstream effectors of RhoA GTPase[75]. The Rho-associated kinase family is comprised of Rho kinase/ROCK2/ROKα and p160 ROCK/ROCK1/ROKβ[76] and are all implicated in the regulation of cytoskeletal organization[77]. Rho kinase transcripts are expressed in the cardiac mesoderm, lateral plate mesoderm, and the neural plate [78]. A pyridine derivative, Y-27632, has been demonstrated to selectively inhibit 160 ROCK[79]. When HH stage 4-8 whole chick embryos or comparable staged mouse embryos are exposed in culture to Y-27632 (as well as antisense to p160 ROCK), precardiac cell migration, cardiac tube formation, and establishment of normal left-right asymmetry are disrupted[78]. In a subsequent study using a reverse genetic approach of cardiac-specific inhibition of Rho family protein, the transgenic embryos showed incomplete looping, lack of chamber demarcation, inhibition of cell proliferation, and lack of trabeculation [80] These described phenotypes of the chick and mouse are similar to those we reported with MMP-2 inhibition or with Pitx2 misexpression and changes in flectin expression[24,25,29]. In addition, Rho kinase via its pleckstrin domain apparently binds directly to NMHC-IIA and –IIB in *in vitro* cosedimentation assays[81]. Inhibition of RhoA/ROCK, using another inhibitor Simvastatin, negatively affects matrix metalloproteinase (MMP) secretion and reduces MMP mRNA levels[82]. We have also shown that MMP-2 is important in a temporal manner in cardiac tube formation, ventral closure, and looping[29]. Vascular endothelial cadherin signals through RhoA/ROCK to regulate cytoskeletal tension and focal adhesions[83]. Additionally, RhoA/ROCK signaling is important for focal adhesion kinase (FAK) activation at focal contacts in cardiomyocytes by cyclic stretch and by its relationship to the cytoskeleton[84]. Phosphorylation of NMHC-IIB regulatory light chain (MLC) regulates NMHC-II activity and this regulation occurs by distinct upstream signals, such as Ca++/calmodulin and the Rho family GTPases. Both signaling pathways are involved in cyclic stretch in cardiomyocytes by their interactions with NMHC-IIB in the cytoskeleton[84]; and Rho-mediated pathway has been shown to perturb looping[78]. It has been shown also that in culture Pitx2a affects actin-myosin interactions through Rho GTPase signaling[85]. That Rho-kinase regulates tissue morphogenesis via nonmuscle myosin has also now been shown during Drosophila development[86]. A Type ID unconventional myosin appears to control left-right asymmetry in Drosophila through the adherens junction as it colocalizes with β-catenin[42]. These reports taken together, suggest that Rho kinase regulates early morphogenetic events associated with cardiac tube formation and looping by altering normal ECM- cell surface-cytoskeletal interactions at focal contacts, and also modulating NMHC-II/actin interactions. These aspects need to be addressed in future experiments in association with looping.

Of interest is that downstream Rho kinase activity affects FAK at focal contacts. These integrin-based focal contacts can act as mechanotransducers of forces associated with stretch as increasing volumes of blood entering the heart during the looping stages to relay information of compressive forces within the cardiac jelly to the myocardial wall. This possibility may relate to our finding that embryos exposed to RGD peptides that inhibit cell binding of extracellular matrix molecules, as fibronectin and collagen, to integrin receptors at focal adhesion sites[87,88], also exhibit randomization of cardiac left-right asymmetry[89]. Normal mechanotransduction signaling would expectedly also be disrupted.

When we analyzed the effects of RGD (GRGDNP; GibcoBRL) peptides on flectin localization in the chick heart during looping, we found a reduced and disorganized expression of flectin together with randomization of looping. In a concentration (10ug/ml to 100 μg/ml) and stage (HH stage 5-8) dependent manner embryos exposed to RGD peptides displayed a down-regulation of flectin (FL) and fibronectin (FN) localization in the heart and basement membrane in the myocardium substratum in comparison to embryos that were treated with similar concentrations of RGA (GRADSP) or RGE (GRGESP) control peptides (Fig. 5). Generally the cardiac ECM matrix compartment was decreased in size with RGD peptide exposure. Chick embryos shown in fig. 5 were exposed to RGD peptides at stage 5/6 and incubated for 24 hrs. After the incubation period, embryos were fixed and doubly immunostained for flectin (FL) and fibronectin (FN). In Fig. 5 A and B embryos exposed to control peptides exhibit normal looping and an extended cardiac jelly compartment. In the RGD-peptide exposed embryos (panels 5C-5H), the anterior heart regions show a relatively normal tubular structure and a high level of flectin in the myocardium (C) and fibronectin (D) at the basal side of the myocardial wall. Fibronectin is also prominent at the midline in association with the ventral floor of the foregut (see white line in D). Unlike in control embryos, little flectin is detectable in the cardiac jelly. After 24 hrs of peptide exposure, the more undifferentiated posterior regions of the heart field at time of exposure, display a much-decreased ECM compartment in the developing heart regions and less flectin expression (panel E) within the cardiac compartments in comparison to fibonectin that generally remains expressed at relatively higher levels. Within the posterior heart areas (panels 5E-5H), flectin becomes almost undetectable, while fibronectin remains expressed. These experiments show that flectin expression in the matrix/basement membrane is significantly decreased, when integrin receptor-mediated cell association with matrix molecules is inhibited. With RGD-exposure both heart development and normal looping are inhibited[87,89].

## NONMUSCLE MYOSIN II AND MECHANOTRANSDUCTION

Myosin is a family of proteins that generates mechanical force by catalyzing hydrolysis of ATP when interacting with actin filaments. Across species more than 10 genes encode myosin heavy chains II. These are divided into two subclasses, the sarcomeric and the nonsarcomeric (smooth and nonmuscle), as based on homologies of primary sequences. In the human genome, there are three genes (MYH) for nonmuscle myosin II defined as NMHC-IIA (MYH9), NMHC-IIB (MYH10), and NMHC-IIC (MYH14). Class II myosins consist of a pair of heavy chains (~200 kDa) and two pairs of light chains (15-20 kDa). Myosin II proteins have been demonstrated to have important functions in diverse cellular contractile and motile processes, including cell migration, muscle contraction, cell division, cell shape changes, cell adhesion, and extracellular matrix remodeling in eukaryotic cells. While a member of the nonmuscle myosin II protein may be present in all eukaryotic cells in higher organisms, they are differentially expressed and appear to have different functions. The functions of NMHC-II proteins appear well conserved across phyla; however, little is yet known about their diverse roles in vertebrate morphogenesis. Biochemical, genetic, and cell biological studies in *Drosophila* and *Dictyostelium,* have demonstrated that nonmuscle myosin II is involved in morphogenesis[74,90,91].

## BIOMECHANICS OF HEART LOOPING

It appears inescapable that interactions involving cytoskeletal components are responsible for biomechanical forces within the heart and dorsal mesocardial folds. These candidate forces include differential growth[29], cardiac jelly swelling[92], active cell shape changes[46], and cytoskeletal contractions[44,47,93]. Some of the biomechanical forces and properties of the heart tube during looping have been described and modeled[94-102].

It is suggested that it is not by chance alone that looping occurs concomitantly with onset of rhythmic heart contractions and initiation of blood flow. Heart tube bending occurs in the mouse embryo at the same time as the cardimyocytes begin to contract and maintain some blood circulation on embryonic day (ED) 8.5-9.0. By ED 10.5 two complete extraembryonic circulations, the yolk sac and the placental, have been established, and are feeding increasing volumes of blood into the heart. By day 14 both the definitive pattern of the prenatal circulatory system and the definitive four-chambered shape of the heart are established. Thus, looping occurs simultaneously with increasing blood flow and the associated stresses on the endocardium. Seemingly, these forces are transmitted through the cardiac jelly to the heart wall to affect cardiomyocyte contractility and rate. Understanding the interrelatedness of blood flow and mechanotransduction involving the cytoskeleton during heart looping will be relevant for a mechanistic understanding of this central defining process of cardiogenesis. Results from early experiments indicated that normally the cardiac jelly is necessary to augment blood flow by its compressive forces[103]. From our own analyses on hemodynamics of the mouse embryonic heart, the valveless tubular heart has diastolic and systolic blood flow without regurgitation and acts as if valves were present[104]. Early myocardial contractions lead to associated forces of pulsatile blood flow. Over time and during looping these forces increase enabling cardiac function. Shear stress and changes in blood flow are reported to have significant roles in cardiac morphogenesis[105,106].

Creation of abnormal flow patterns, such as by use of a venous clip were reported to cause malformations similar to those seen in knockouts for genes such as endothelin-1 (ET-1)[107,108,109]. Shear stress is defined as the product of the viscosity of blood and the velocity gradient (which is essentially an indicator of the distribution of velocities throughout the cross-section of the vessel). For a simple tube geometry, the shear stress is proportional to flow rate and inversely proportional to $R^3$, where R is the radius of the vessel. Based on this, one would expect that higher stress would be found in areas of constriction or narrowing, and lower in larger structures such as the developing atria. Based on these assumptions, the pattern of expression of shear stress responsive genes Kruppel-like factor (KLF-2), ET-1 and nitric oxide synthase (NOS-3) have been examined in the developing heart. It was shown that KLF-2 is expressed in areas expected to have higher shear stress, and changes in ET-1 and NOS-3 expression through development appear to be related to changes in shear stress based on the changes in size and shape of the developing structures[110]. We have also detected a high level of flectin expression in a region of high shear stress in association with a narrowing of the cardiac tube within the sinoatrial region (Fig. 4). Shear stress is highly dependent on geometry. For example, there will be an uneven distribution of shear stress along the walls of a curved vessel. For a given geometry, there are also differences in shear stress distributions and cellular responses to shear stresses in pulsatile flow, as compared to steady flow. Thus, both morphometric changes and timing of heart contractions may play a role in determining stress distributions, and thus further morphogenetic changes, due to responses to stress to tightly coordinate heart structure and function[31,56].

It is difficult at best to measure shear stress at any particular location. Thus, precise mapping of shear stress is not feasible. An attempt has been made to determine more precisely flow patterns in Stage 10 and 11 human embryo hearts by converting images to computational fluid dynamic (CFD) models[111]. CFD seeks numerical approximations to equations of motion that represent the physics of blood flow, providing maps of quantities such as velocity and pressure throughout the model. Shear stresses can also be computed from these data. The simulations did indeed predict features of observed blood flow patterns. Digital particle image velocimetry and optical coherence tomography (OCT) are techniques with the potential to provide quantitative information regarding blood flows[112,113], that could be used to ensure validity of the simulations and increase confidence in the calculations of the shear stresses that cannot be measured directly.

The biomechanical properties of the cardiac jelly and myocardium have been explored[96]. The cardiac jelly in HH stage 12 hearts was found to be “softer” than the vitreous body of the eye, and experiences compressive stresses due to pressure exerted within the heart tube and the action of the myocardium. During looping, the inner curvature of the heart tube is stiffer than the outer curvature [114]. In later stages, the myocardium becomes increasingly stiffer with age. This occurs as sarcomeres are formed and the tissue becomes better organized[96], but stiffening also occurs when pressure in the developing heart increases (for example, during chamber septation)[115]. Material properties of the dorsal aorta and systemic arteries also adapt to increased pressure[116]. It is unclear whether changes in material properties are causative or merely consequences of heart looping and development. What is clear is that there are complex interactions between the fluid mechanics and solid mechanics of the developing heart that warrant further study.

Transmission of myocardial contractile forces through the cardiac jelly may involve cell focal adhesions with the ECM molecules of the basal lamina. The generation of forces to accommodate increasing stresses thus can be linked with the myocardial cytoskeleton via cell processes that extend and

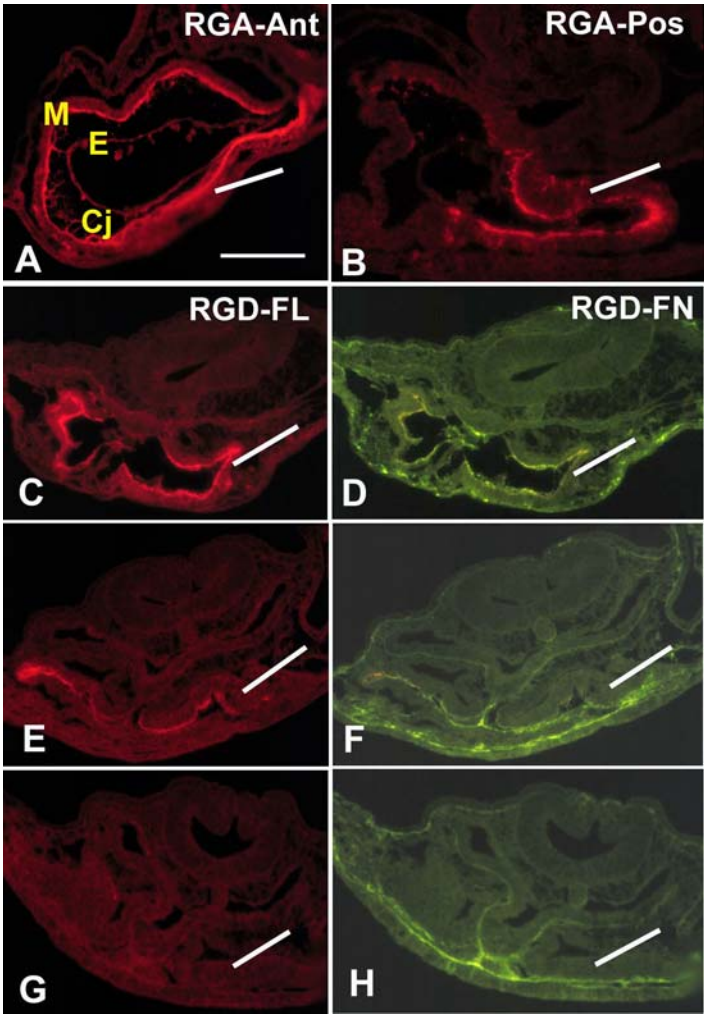

**FIGURE 4**. Exposure of HH stage 5 chick embryos to RGD peptides results in an inhibition of normal heart looping and a decrease of FL expression, as well as a decrease in the cardiac jelly compartment of the developing heart. Panels A and B show an embryo that was exposed to control RGA-containing peptides. The heart displays normal FL expression in the basal side of the myocardium and in the cardiac jelly as seen in a section through the anterior (Ant) part of the looping heart (A), and in a more posterior (Pos) section through the same heart that continues to show FL expression primarily in the left side of the heart tube (B). Panels C–H depict results of embryonic exposure to RGD-containing peptides. Panels C, E, and G show an anterior to posterior decrease of FL within the developing heart (white lines point to developing cardiac tissue) on exposure to the RGD peptide in relation to FN in panels D, F, and H that continues to be expressed throughout the heart regions. It is also evident that there is a decrease in ECM production of the cardiac jelly with RGD-peptide inhibition. Magnification bar = 120 µm.

interact with the ECM components associated with the basal layer of the myocardium. Similarly, monocilia have been shown on embryonic endocardial-endothelial cells that are postulated to function as fluid shear stress sensors[117]. The likelihood that cardiomyocyte cell extensions are integrin mechanosensor related is based on several observations: Already early in development cardiac cells express integrin β1 subunit[88]. Myocardial cells are associated with FN in the basement membrane (Fig. 5), and the myocardial cell processes do not express acetylated tubulin (Fig. 6), that is detected in true microcilia. Therefore we suggest that basal myocardiocyte cell processes may act as mechanosensors to mediate the exchange of inside-out and outside-in information through transmembrane integrin receptor-mediated interactions with extracellular matrix molecules within the myocardial basal lamina. This appears also to be the case in the zebrafish heart where an integrin-linked kinase was shown to be a component of the cardiac mechanical stretch sensor to control contractility in the zebrafish heart[118].

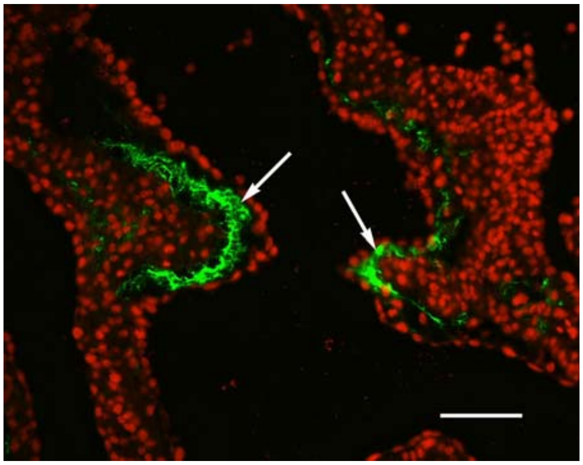

**FIGURE 5.** Flectin (green fluorescence; see arrows) is evident in the ECM at HH stage 17 in the sinus venosus region of the chick heart where a narrowing of the heart tube is observed. This is a region where a high shear stress of blood flow would be expected. Magnification bar = 50 um.

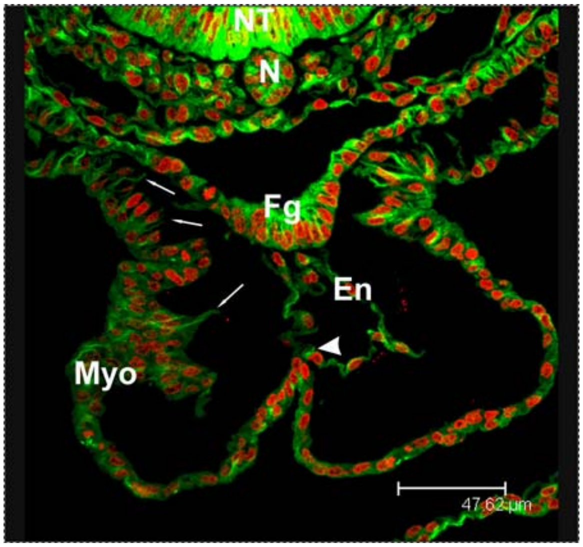

**FIGURE 6**. HH stage 11 chick heart immunostained for acetylated tubulin. Myocardial cell processes extending into the cardiac jelly (see arrows) do not express acetylated tubulin that is observed usually in microcilia (arrowhead) and that are associated with endocardial-endothelial cells. This lack of tubulin expression in cardiomyocyte cell extensions into the ECM of the basal lamina and the RGD experiments suggest that myocardial mechanosensing of forces associated with blood flow would most likely be through integrin-mediated signaling. NT, neural tube; N, notochord; Fg, foregut; Myo, myocardium; En, endocardium. Magnification bar = 47.62 µm.

The presence of mechanoreceptors would allow signal transduction and feedback from the myocardial wall and endocardium to accommodate blood flow increases, as well as possibly to regulate completion of looping. It appears logical to deduce that evolution has enabled the organization of the cytoskeleton for both generation of force and the ability to undergo deformation while adapting to force.

## ACKNOWLEDGMENTS

The research in relation to looping, FL, and NMHC-II studies was generously supported by grants from American Heart Association Established Investigator Grant and from the Suncoast Cardiovascular Research and Education Foundation founded by Helen Harper Brown (to KKL). The following collaborations are greatly appreciated: Dr. Isao Shiraishi at the Kyoto Prefectural University of Medicine in Japan for generation of Fig. 3; Dr. Guoying Huang of Shanghai Medical University for generation of the RGD data shown in Fig. 4 during his postdoctoral training in my lab (KKL); and Dr. Beerend Hierck and his student Kim Van der Heiden at the Leiden University Medical Center in the Netherlands for preparation of Figs. 5 and 6.
